# Successful treatment of invasive gastric mucormycosis in a patient with alcoholic liver cirrhosis: A case report

**DOI:** 10.3892/etm.2014.1753

**Published:** 2014-06-03

**Authors:** SEUNG HYOUNG LEE, YOUNG GIL SON, SOO SANG SOHN, SEUNG WAN RYU

**Affiliations:** Department of Surgery, Dong San Medical Center, Keimyung University School of Medicine, Daegu 700-712, Republic of Korea

**Keywords:** mucormycosis, gastrointestinal mucormycosis, cirrhosis

## Abstract

Gastrointestinal (GI) mucormycosis is a rare and life-threatening invasive fungal infection. GI mucormycosis occur in all parts of the alimentary tract, with the stomach being the most common site. Diabetes mellitus and other types of conditions associated with immunodeficiency, including hematologic malignancies, solid organ transplantation and glucocorticoid therapy, are risk factors for GI mucormycosis. There are few studies reporting cases of gastric mucormycosis in patients with liver cirrhosis, and even fewer reporting the successful treatment of invasive gastric mucormycosis in a patient with liver cirrhosis. This study presents a case of invasive gastric mucormycosis in a patient with liver cirrhosis, which was treated successfully by prompt diagnosis, metabolic support, surgical debridement of involved tissues and antifungal therapy.

## Introduction

Mucormycosis is a rare and life-threatening invasive fungal infection caused by fungi of the Zygomycetes class and Mucorales order. *Rhizopus*, *Mucor* and *Rhizomucor* are the genera of Mucorales that are most frequently identified in human infections (1). Based on the clinical presentation and involvement, mucormycosis is classified as six major forms, namely, rhinocerebral, pulmonary, cutaneous, gastrointestinal (GI), disseminated and miscellaneous (2,3), with rhinocerebral and pulmonary being the common forms. GI mucormycosis is rare, accounting for only 7% of all cases; however, the mortality rate is as high as 85% (1). GI mucormycosis may occur in any part of the alimentary tract, with the stomach being most commonly involved, followed by the colon and ileum (4,5). GI mucormycosis occurs more frequently in patients with diabetes mellitus and other conditions associated with immunodeficiency, including hematologic malignancies, solid organ transplantation, glucocorticoid therapy, chronic renal failure, liver cirrhosis and malnutrition in infants and children (6–8). There are only a few reports of successfully treated invasive gastric mucormycosis due to its high mortality rate (9–11). Gastric mucormycosis in patients with liver cirrhosis has rarely been reported (12). Therefore, the present study reports a case of successfully treated invasive gastric mucormycosis in a patient with alcoholic liver cirrhosis, and reviews the associated studies.

## Case Report

**Figure 1:**
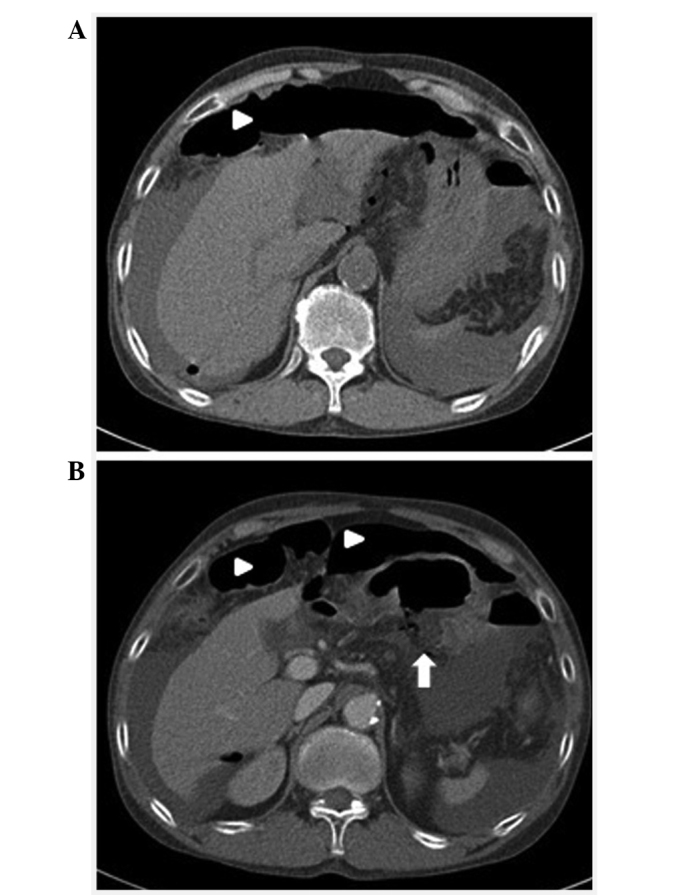
Computed tomography (CT) scans of the abdomen. (A) CT scan revealed pneumoperitoneum (arrowheads), fluid collection of abdomen and shrinkage of the liver. (B) CT scan revealed a gastric ulcer perforation at the posterior wall of the antrum (arrows).

A 55-year-old male with alcoholic liver cirrhosis was referred to the emergency department of Keimyung University Dongsan Medical Center (Daegu, Republic of Korea) from a local hospital, complaining of severe, constant pain throughout the whole abdomen for 6 h. According to the patient’s past medical history, the patient had been treated with medication for gastric ulcers for one month immediately prior to admission to Keimyung University Dongsan Medical Center. The patient also had liver cirrhosis due to excessive ingestion of alcohol for 35 years. Upon arrival at the emergency department, the patient appeared acutely ill and mildly dehydrated. However, the vital signs were within normal range as sufficient volumes of fluid had been administered at the local hospital. The patient did not exhibit jaundice, hepatosplenomegaly or signs of chronic liver disease. Abdominal examination revealed severe tenderness and rebound tenderness throughout the whole abdomen with rigidity and hypoactive bowel sounds. Furthermore, the laboratory data on arrival noted reduced levels of hemoglobin, measuring 3.6 g/dl (normal, 12–18 g/dl), and the other laboratory tests were normal. An abdominal computed tomography (CT) scan revealed pneumoperitoneum and hemoperitoneum due to gastric ulcer perforation (Fig. 1). Subsequently, the patient underwent an emergency laparotomy.

**Figure 2:**
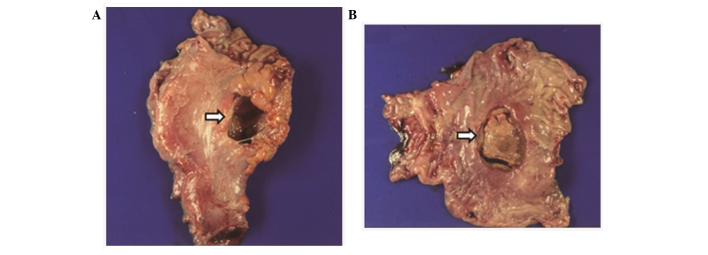
A gross surgical specimen from the gastrectomy shows a large defect of the stomach (arrows). (A) View of serosal surface. (B) View of the mucosal surface.

The surgical findings showed an unusually large perforation, measuring 5.0×3.7 cm in gastric angle (Fig. 2), with ~2,000 ml ascitic fluid collection in the abdominal cavity which was not bloody. Severe cirrhotic changes of the liver were also noted. A subtotal gastrectomy and gastrojejunostomy with massive irrigation in the abdominal cavity was performed. The patient was then transferred to the intensive care unit for further management.

**Figure 3:**
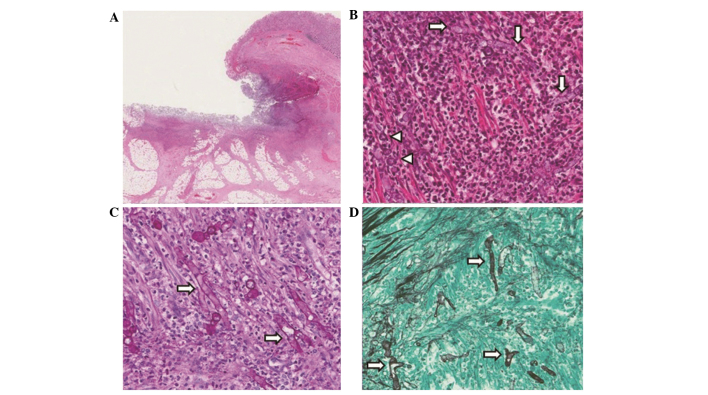
Microscopic findings of the gastric ulcer. (A) H&E staining showed a deep gastric ulcer covered with exudates was observed (staining, H&E; magnification, ×40). (B) H&E staining showed numerous fungal hyphae (arrows) and yeasts (arrowheads) were admixed with acute inflammatory cells and ulcer debris (staining, H&E; magnification, ×200). (C) PAS staining showed numerous non septated fungal hypae (arrows) with PAS positive thick walls (staining, PAS; magnification, ×200) (D) GMS staining revealed numerous broad, irregular and non septated fungal hypae (arrows) (staining, GMS; magnification, ×200). H&E, Hematoxylin and eosin; PAS, periodic acid Schiff; GAS, Grocott’s methenamine silver.

On the fourth day in the hospital, gastric mucormycosis was cited in the preliminary pathology report. Antifungal therapy was promptly initiated using amphotericin B deoxycholate (0.6 mg/kg/day). However, following administration of the agent for 3 h, the vital signs became unstable (blood pressure 80/60 mmHg and tachycardia), so the amphotericin B deoxycholate was replaced with liposomal amphotericin B (5 mg/kg/day). On the seventh day in hospital, the pathologic diagnosis was confirmed as invasive gastric mucormycosis (Fig. 3).

Hepatotonics, fresh frozen plasma, platelets and albumin were supplied following the surgery due to persistent thrombocytopenia, hypocoagulopathy and hypoalbuminemia caused by the underlying liver cirrhosis. Diuretics were also administered to manage the large amount of ascites, of which ~800–1,000 ml was drained for 10 days.

On the twenty-first day in hospital, treatment with liposomal amphotericin B was ceased. All vital signs and laboratory data were normal and the patient was in a satisfactory condition without antifungal treatment. On the twenty-fourth day in hospital, the patient was discharged to return home with a good health status. Written informed consent was obtained from the patient. The study was approved by the ethics committee of Dong San Medical Center, Keimyung University School of Medicine, Daegu, Republic of Korea.

## Discussion

Mucormycosis is an uncommon type of fungal infection, most frequently occurring in immunocompromised patients and those with diabetes mellitus (1). Mucormycosis was first reported as a cause of human disease by Paultauf in 1885 (13). Mucormycosis refers to any fungal infections caused by fungi of the Mucorales order, which belongs to the Zygomycetes class. Certain Zygomycetes genera, including *Rhizopus*, *Mucor* and *Rhizomucor,* are frequently observed in human infection and *Saksenaea*, *Cunninghamella*, *Absidia* and *Apophysomyces* are the genera less commonly identified in infections (14,15). The diseases produced by the aforementioned organisms are almost identical and, thus far, the diagnosis and therapy of mucormycosis has not been influenced by identification of the specific species of the pathogens. Thus, visualization of the characteristic hyphae in sections of tissue pathologically is essential for the definitive diagnosis of mucormycosis. The hyphae of Mucorales are different from those of other types of mold. The hyphae of Mucorales in tissue are irregular-shaped, broad (5–20 μm in diameter) and have rare septation (14).

The clinical manifestation of mucormycosis has been traditionally divided into six major forms, namely, rhinocerebral, pulmonary, cutaneous, GI, disseminated and miscellaneous (including endocarditis, osteomyelitis and renal infection) (2,3). The common sites of mucormycosis have been reported as the sinuses (39%), lungs (24%), skin (19%), brain (9%), GI tract (7%) and the kidneys (2%), in addition to disseminated infection (3%) (1). The mode of transmission is mainly airborne, thus the sinuses and pulmonary manifestations are more frequent. Although it is unusual, mucormycosis of the GI tract may occur as the result of ingestion of spores in food, including fermented milk, dried bread products, and spore-contaminated herbal and homeopathic remedies (7,16).

Patients with GI mucormycosis may present abdominal pain and distension, fever, and hematemesis and hematochezia due to GI bleeding. The GI lesions are necrotic ulcers that may lead to bowel bleeding and perforation, peritonitis, sepsis and hemorrhagic shock (11,17). Therefore, the prognosis for all patients with GI mucormycosis is poor and the majority of patients are often diagnosed following mortality caused by the infection (1).

GI mucormycosis may occur in any part of the alimentary tract, with the most common site being the stomach (58%), followed by the colon (32%), ileum and esophagus (4,5). Gastric mucormycosis is primarily observed in patients with diabetes mellitus, solid organ transplantation, glucocorticoid use, liver cirrhosis, renal failure, deferoxamine administration, prematurity and malnutrition (6,18). In addition, alcoholism appears to be a risk factor of gastric mucormycosis. Following a review of the case in the present study, three previous studies on individual cases of gastric mucormycosis in patients with alcoholism were identified (10,19,20). Ho *et al* (20) reported that the ingestion of fungal spores and their germination may be harmful to a patient with alcoholism as ethanol may disrupt the activation of macrophages and dendritic cells, which play crucial roles in the immune reaction that eliminates fungal spores.

Also, as evidenced in the case in the present study, chronic alcohol abuse may lead to liver cirrhosis. Liver cirrhosis itself lowers systemic immunity, resulting in an increased likelihood of gastric mucormycosis, and also causes coagulation dysfunction and hypoalbuminemia, increasing the risk of post-operative complications and resulting in an increased mortality rate.

Management of gastric mucormycosis includes prompt diagnosis, metabolic support, elimination of predisposing factors, aggressive surgical debridement of involved tissues and antifungal therapy. Prompt and extensive surgical debridement is, possibly, the most important option for treatment, and the aim of the surgery should be to remove all necrotic tissue. Also, the early initiation of antifungal therapy is important for patients who have gastric mucormycosis (8,17). Previously, a study has reported that early initiation of amphotericin B therapy improves the outcome of infection with mucormycosis (21).

Intravenous amphotericin B (including its deoxycholate salt, lipid derivatives and liposomal formulations) is the standard antifungal therapy for gastric mucormycosis. The starting dose of amphotericin B deoxycholate is 1–1.5 mg/kg/day and the usual starting dose of amphotericin B lipid complex or liposomal amphotericin B is 5 mg/kg/day. The majority of clinicians use a lipid formulation of amphotericin B or liposomal amphotericin B in order to deliver a high dose with less nephrotoxicity. The duration of amphotericin B therapy has not yet been defined, but is guided by the resolution of associated symptoms and findings (usually 6–8 weeks). Furthermore, a novel broad-spectrum oral azole agent named posaconazole has a potential role in the treatment of mucormycosis. Posaconazole is used as a step-down therapy for patients who have responded to amphotericin B and rarely as salvage therapy for patients who do not respond to or tolerate amphotericin B (22,23). In the case in the present study, only liposomal amphotericin B was used for 21 days without posaconazole step-down therapy and no abnormal signs or symptoms were observed after treatment with the antifungal agents ceased. Therefore, further studies are required to determine the definite criteria and duration of the use of antifungal agents to treat gastric mucormycosis.

To the best of our knowledge, this is the first study to report the successful treatment of gastric mucormycosis, revealed by gastric ulcer perforation, in a patient with alcoholic liver cirrhosis. To achieve the successful treatment of gastric mucormycosis in a patient with alcoholic liver cirrhosis, we consider that prompt diagnosis, aggressive surgical debridement of the involved tissues, early use of amphotericin B and sufficient metabolic support to prevent hepatic failure are essential and ultimately improve the survival rate. Furthermore, further studies on gastric mucormycosis in patients with liver cirrhosis are required to design a treatment strategy to minimize the post surgery complications and improve the survival rate.
